# Risk factors for and clinical outcomes of carbapenem non-susceptible gram negative bacilli bacteremia in patients with acute myelogenous leukemia

**DOI:** 10.1186/s12879-020-05131-2

**Published:** 2020-06-09

**Authors:** Dong Hoon Shin, Dong-Yeop Shin, Chang Kyung Kang, Suhyeon Park, Jieun Park, Kang Il Jun, Taek Soo Kim, Youngil Koh, Jun Shik Hong, Pyoeng Gyun Choe, Wan Beom Park, Nam-Joong Kim, Sung-soo Yoon, Inho Kim, Myoung-don Oh

**Affiliations:** 1 Department of Internal Medicine, Seoul National University College of Medicine, Daehak-ro 101, Jongro-gu, Seoul, 03080 Republic of Korea; 2 Cancer Research Institute, Seoul National University College of Medicine, Daehak-ro 101, Jongro-gu, Seoul, 03080 Republic of Korea; 3 Department of Laboratory Medicine, Seoul National University College of Medicine, Daehak-ro 101, Jongro-gu, Seoul, 03080 Republic of Korea

**Keywords:** Carbapenem non-susceptible, Gram negative bacilli, Bacteremia, Acute myelogenous leukemia, Risk factors

## Abstract

**Background:**

Carbapenem is frequently used when gram negative bacilli (GNB) bacteremia is detected especially in neutropenic patients. Consequently, appropriate treatment could be delayed in GNB bacteremia cases involving organisms which are not susceptible to carbapenem (carba-NS), resulting in a poor clinical outcomes. Here, we explored risk factors for carba-NS GNB bacteremia and its clinical outcomes in patients with acute myelogenous leukemia (AML) that underwent chemotherapy.

**Methods:**

We reviewed all GNB bacteremia cases that occurred during induction or consolidation chemotherapy, over a 15-year period, in a tertiary-care hospital.

**Results:**

Among 489 GNB bacteremia cases from 324 patients, 45 (9.2%) were carba-NS and 444 (90.8%) were carbapenem susceptible GNB. Independent risk factors for carba-NS GNB bacteremia were: carbapenem use at bacteremia onset (adjusted odds ratio [aOR]: 91.2; 95% confidence interval [95%CI]: 29.3–284.1; *P* < 0.001); isolation of carbapenem-resistant *Acinetobacter baumannii* (aOR: 19.4, 95%CI: 3.4–112.5; *P* = 0.001) in the prior year; and days from chemotherapy to GNB bacteremia (aOR: 1.1 per day, 95%CI: 1.1–1.2; *P* < 0.001). Carba-NS bacteremia was independently associated with in-hospital mortality (aOR: 6.6, 95%CI: 3.0–14.8; P < 0.001).

**Conslusion:**

Carba-NS organisms should be considered for antibiotic selection in AML patients having these risk factors.

## Background

Bloodstream infection is a common and serious infectious complication in patients with hematologic malignancy, having the crude mortality rate of 12–42% [1–3]. Above all, bloodstream infections caused by gram negative bacilli (GNB) are a major cause of morbidity and mortality in patients with acute myelogenous leukemia (AML) that undergo induction or consolidation chemotherapy [4, 5]. Patients with AML typically experience a prolonged period of neutropenia, which increases the risk of bacteremia and leads to poor outcome [3, 5]. Since inappropriate empiric treatment is a well-known risk factor for a fatal outcome [6–8], early administration of an effective antibiotic is crucial to treat this deadly condition.

Carbapenems are frequently used as an empirical treatment for a broad spectrum of bacteria in patients that exhibit clinical deterioration in febrile neutropenia. Consequently, appropriate treatment might be delayed when pathogens are not susceptible to carbapenem (carba-NS), which could lead to a poor clinical outcome [9, 10]. A few studies have investigated risk factors for carba-NS GNB bacteremia and its clinical outcomes [9, 10]. However, no study has elucidated risk factors in patients with AML that receive chemotherapy.

We aimed to explore the risk factors for carba-NS GNB bacteremia and its clinical outcomes in patients that underwent induction or consolidation chemotherapy for AML.

## Methods

### Study design

This retrospective case-control study, conducted in Seoul National University Hospital, included all adult patients (aged ≥ 18 years) with AML that contracted GNB bacteremia during induction or consolidation chemotherapy, from January 2000 to December 2015. Clinical and microbiological characteristics were compared between cases of carbapenem susceptible (carba-S) and cases of carba-NS GNB bacteremia. We also examined the association between carba-NS and in-hospital mortality.

### Clinical characteristics

We retrieved clinical data from electronic medical records related to patient characteristics, including: age, sex, purpose of chemotherapy (induction or consolidation), history of diabetes mellitus, history of a resistant organism colonization in the preceding year (namely, vancomycin-resistant enterococci (VRE), extended-spectrum β-lactamase-producing enterobacteriaceae (ESBL), carbapenem-resistant *Pseudomonas aeruginosa*, and carbapenem-resistant *Acinetobacter baumannii* (CRAB)), history of GNB bacteremia in the prior year, primary site of infection, the acute severity of sepsis measured with the Pitt bacteremia score [11, 12], the presence of septic shock, antibiotics used at bacteremia onset and those used empirically before the antibiotic susceptibility results become available, and the hospitalization duration before the GNB bacteremia onset. We also collected data on all-cause mortality within 30 days after bacteremia onset, in-hospital mortality, and GNB-bacteremia-attributed mortality.

### Definitions

GNB bacteremia was defined as the isolation of a GNB in at least one blood culture bottle [13]. All kinds of blood GNB isolates were regarded as pathogens. When multiple bacteremia events had occurred in a patient during a single chemotherapy treatment, each bacteremia caused by a different organism was considered a different case. Antibiotic treatments were categorized into four groups: none; antibiotics without antipseudomonal activity; antipseudomonal agents, but not carbapenem; and carbapenem. For example, non-antipseudomonal agents included cefazolin or ceftriaxone, and antipseudomonal antibiotics that were not carbapenem included piperacillin/tazobactam or cefepime. Empirical antibiotics were considered inappropriate, when the isolate was not susceptible to the selected antibiotics in vitro. Septic shock was defined as sepsis associated with systolic blood pressure below 90 mmHg or 40 mmHg below the baseline systolic blood pressure; or a requirement for a vasopressor to maintain blood pressure, despite adequate fluid resuscitation [7]. GNB bacteremia-attributed mortality was defined as positive blood culture for GNB at the time of death or a persistent focus of GNB infection associated with clinical signs of sepsis [14, 15].

### Microbiological analyses

BacT/Alert FA and FN bottles (bioMe’rieux, Durham, North Carolina) were used for all blood cultures. Antimicrobial susceptibility was identified with VITEK 2 (bioMe’rieux, Marcy L’etoil, France), according to the criteria provided in the guidelines of the Clinical and Laboratory Standards Institute [16]. A bacterium was regarded as a carba-NS strain when it showed intermediate susceptibility or resistance to at least one carbapenem in vitro [16].

### Sensitivity analyses

Sensitivity analyses were performed to account for potential confounders of risk factors for carba-NS GNB bacteremia or in-hospital mortality. These subgroup analyses were performed after excluding breakthrough GNB bacteremia cases that occurred during carbapenem use, since carbapenem use had been suspected to be strongly associated with the occurrence of carba-NS GNB bacteremia. Risk factors for carba-NS GNB bacteremia and those for in-hospital mortality were examined in the subgroup similar to the methods described above.

### Statistical analysis

The Student’s *t* test was used to compare continuous variables, and the chi-squared test or Fisher’s exact test were used to compare categorical variables. All the variables were included in the multivariate analysis using a backward stepwise logistic regression, with *P* = 0.05 as the cut-off value for removing variables. Among parameters that represented the length of hospital stay and acute severity of infection, only a time interval between starting date for chemotherapy and the date of positive blood culture for GNB and Pitt bacteremia score were included in the multivariate analysis to avoid multicollinearity, respectively. Multicollinearity was checked using variance inflation factor with 5 as the cutoff. Risk factors for in-hospital mortality or GNB bacteremia-attributed mortality were also analyzed with a multivariate regression analysis, as described above. All *P* values < 0.05 were considered statistically significant. All analyses were performed with PASW for Windows (version 25.0; SPSS Inc., Chicago, IL, USA).

## Results

### GNB bacteremia cases

During the study period, 929 patients underwent 1562 episodes of induction or consolidation chemotherapy. Among those patients, we identified 489 GNB bacteremia cases in 324 patients since 116 patients (35.8%) suffered from GNB bacteremia more than once. Among those cases, 122 (24.9%), 81 (16.6%), and 286 (58.5%) occurred during induction, re-induction, and consolidation chemotherapy, respectively. Of the total GNB bacteremia cases, 45 (9.2%) were carba-NS GNB and 444 (90.8%) were carba-S GNB.

### Clinical characteristics of patients with carba-NS GNB bacteremia

The clinical characteristics of the two patient groups are described in Table 1. The age of patients with carba-NS GNB bacteremia was significantly higher than that of patients with carba-S GNB bacteremia (mean ± standard deviation: 56.4 ± 13.8 vs. 51.0 ± 14.8; *P* = 0.019). Male tended to be more frequent in carba-S group (59.2%) than in carba-NS group (44.4%; *P* = 0.056). The most frequent carba-NS isolates were *Stenotrophomonas maltophilia* (23 cases, 51.1%), *Pseudomonas aeruginosa* (11 cases, 24.4%), and *Acinetobacter baumannii* (10 cases, 22.2%). Among the carba-NS GNB bacteremia cases, 10 (22.2%) were polymicrobial infections.

**Table 1:** Clinical characteristics of patients with carba-S and carba-NS GNB bacteremia

Variables	Carba-S (*n* = 444)	Carba-NS (*n* = 45)	*P*
Age, mean (± SD)	51.0 (± 14.8)	56.4 (± 13.8)	0.019
Male	263 (59.2)	20 (44.4)	0.056
Chemotherapy			< 0.001
Induction or re-induction	170 (38.3)	33 (73.3)	
Consolidation	274 (61.7)	12 (26.7)	
Diabetes mellitus	123 (27.7)	13 (28.9)	0.866
Isolation of resistant organism in the prior 1 year			
VRE	33 (7.4)	11 (24.4)	0.001
ESBL	22 (5.0)	7 (15.6)	0.011
CRPA	9 (2.0)	2 (4.4)	0.268
CRAB	3 (0.7)	6 (13.3)	< 0.001
History of GNB bacteremia in the prior 1 year	146 (32.9)	21 (46.7)	0.063
Presence of preceding bacteremia during the hospitalization	42 (9.5)	19 (42.2)	< 0.001
Primary foci of infection			
Intra-abdominal infection	67 (15.1)	5 (11.1)	0.473
Central line associated infection	52 (11.7)	9 (20.0)	0.086
Urinary tract infection	8 (1.8)	0 (0.0)	1.000
Pneumonia	6 (1.4)	0 (0.0)	1.000
Others	9 (2.0)	0 (0.0)	0.610
Unknown	303 (68.2)	31 (68.9)	0.929
Pitt score, median (IQR)	1.6 (0.0–2.0)	2.2 (0.0–4.0)	0.171
Septic shock	92 (20.7)	10 (22.2)	0.813
Microorganism			
*E.coli*	196 (44.1)	0 (0.0)	< 0.001
*Klebsiella* spp.	127 (28.6)	2 (4.4)^a^	< 0.001
Other enterobacteriaceae	75 (16.9)	1 (2.2)^a^	0.010
*Pseudomonas* spp.	36 (8.1)	11 (24.4)^b^	0.002
*S. maltophilia*	0 (0.0)	23 (51.1)^c^	< 0.001
*Acinetobacter* spp.	4 (0.9)	10 (22.2)^d^	< 0.001
Others	32 (7.2)	3 (6.7)^e^	0.561
Antibiotics at the onset of GNB bacteremia			< 0.001
None	252 (56.8)	4 (8.9)	
Agents without antipseudomonal activity	40 (9.0)	2 (4.4)	
Antipseudomonal agents, not carbapenem	147 (33.1)	14 (31.1)	
Carbapenem	5 (1.1)	25 (55.6)	
Inappropriate empiric antibiotics	12 (2.7)	28 (62.2)	< 0.001
Hospital stay			
Days from chemotherapy to GNB bacteremia, median (IQR)	14 (11–16)	22 (16–29)	< 0.001
Hospital days to GNB bacteremia, median (IQR)	22 (14–20)	39 (23–53)	< 0.001
Clinical outcomes			
30-day mortality	25 (5.6)	16 (35.6)	< 0.001
In-hospital mortality	42 (9.5)	21 (46.7)	< 0.001

*Carba-S* carbapenem susceptible, *Carba-NS* carbapenem non-susceptible, *SD* standard deviation, *VRE* vancomycin resistant enterococci, *ESBL* extended-spectrum β-lactamase-producing enterobacteriaceae, *CRPA* carbapenem resistant *Pseudomonas aeruginosa*, *CRAB* carbapenem resistant *Acinetobacter baumannii*, *GNB* gram negative bacilli, *IQR* interquartile range

^a^ One case was mixed GNB bacteremia case

^b^ Three cases were mixed GNB bacteremia cases

^c^ Eight cases were mixed GNB bacteremia cases

^d^ Two cases were mixed GNB bacteremia cases

^e^ Namely, two and one cases were *Elizabethkingia meningoseptica*, and *Burkholderia cepacia*, respectively

Compared to the carba-S group, the carba-NS group showed significantly higher percentages of induction or re-induction chemotherapy, rather than consolidation (*P* < 0.001); isolation of VRE (*P* = 0.001), ESBL (*P* = 0.011), or CRAB (*P* < 0.001) in the prior year; preceding bacteremia during the hospitalization (*P* < 0.001); and carbapenem use at bacteremia onset (*P* < 0.001). In addition, the longer period from chemotherapy to bacteremia onset (*P* < 0.001), and the longer hospital stay (*P* < 0.001) were associated with carba-NS cases.

### Independent risk factors for carba-NS GNB bacteremia

A multivariate logistic analysis identified three independent risk factors for carba-NS GNB bacteremia. These risk factors were: carbapenem use at the onset of bacteremia (adjusted odds ratio [aOR]: 91.2, 95% confidence interval [95%CI]: 29.3–284.1, *P* < 0.001); the isolation of CRAB in the prior year (aOR: 19.4, 95%CI: 3.4–112.5, *P* = 0.001); and days from chemotherapy to GNB bacteremia (aOR: 1.1 per day, 95%CI: 1.1–1.2, *P* < 0.001; Table 2).

**Table 2:** Independent risk factors for carba-NS GNB bacteremia^a^

Variables	aOR (95% CI)	*P*
Carbapenem use at the onset of GNB bacteremia	91.2 (29.3–284.1)	< 0.001
Isolation of CRAB in the prior 1 year	19.4 (3.4–112.5)	0.001
Days from chemotherapy to GNB bacteremia	1.1 (1.1–1.2)	< 0.001

*aOR* adjusted odds ratio, *CI* confidence interval, *GNB* gram negative bacilli, *CRAB* carbapenem resistant *Acinetobacter baumannii*

^a^ Following variables were included in the backward stepwise logistic regression: age, induction or re-induction chemotherapy, carbapenem use at bacteremia onset, isolation of vancomycin-resistant enterococci, extended-spectrum β-lactamase-producing enterobacteriaceae, or CRAB in the prior year, preceding bacteremia during the hospitalization, and days from chemotherapy to GNB bacteremia

### Independent risk factors for mortality

Carba-NS cases were significantly frequent in in-hospital mortality cases than in survival ones (21/63, 33.3% in in-hospital mortality vs. 24/426, 5.6% in survival; *P* < 0.001, Table 3). Carba-NS GNB bacteremia was independently associated with in-hospital mortality (aOR: 6.6, 95%CI: 3.0–14.8, *P* < 0.001) after adjusting with induction or re-induction chemotherapy rather than consolidation (aOR: 3.5, 95%CI: 1.7–7.0, *P* = 0.001), the isolation of VRE in the prior year (aOR: 4.3, 95%CI: 1.8–10.2, *P* = 0.001), pneumonia as a primary focus (aOR: 32.7, 95%CI: 5.1–208.5, *P* < 0.001), or the Pitt score (aOR: 1.5 per score point, 95%CI: 1.3–1.7, *P* < 0.001).

**Table 3:** Risk factors for in-hospital mortality

Variables	Survival (*N* = 426)	In-hospital mortality (*N* = 63)	Univariate		Multivariate	
			OR (95%CI)	*P*	aOR (95%CI)	*P*
Age, mean (± SD)	50.9 (± 14.8)	55.4 (± 14.2)	1.0 (1.0–1.0)	0.025	–	–
Male	250 (58.7)	33 (52.4)	0.8 (0.5–1.3)	0.344	–	–
Chemotherapy						
Induction or re-induction	158 (37.1)	45 (71.4)	4.2 (2.4–7.6)	< 0.001	3.5 (1.7–7.0)	0.001
Consolidation	268 (62.9)	18 (28.6)	–	–	–	–
Diabetes mellitus	113 (26.5)	23 (36.5)	1.6 (0.9–2.8)	0.099	–	–
Isolation of resistant organism in the prior 1 year						
VRE	27 (6.3)	17 (27.0)	5.5 (2.8–10.8)	< 0.001	4.3 (1.8–10.2)	0.001
ESBL	21 (4.9)	8 (12.7)	2.8 (1.2–6.6)	0.023	–	–
CRPA	8 (1.9)	3 (4.8)	2.6 (0.7–10.1)	0.158	–	–
CRAB	4 (0.9)	5 (7.9)	9.1 (2.4–34.8)	0.003	–	–
History of GNB bacteremia in the prior 1 year	136 (31.9)	31 (49.2)	2.1 (1.2–3.5)	0.007	–	–
Presence of preceding bacteremia during the hospitalization	42 (9.9)	19 (30.2)	3.9 (2.1–7.4)	< 0.001	–	–
Primary foci of infection						
Intra-abdominal infection	66 (15.5)	6 (9.5)	0.6 (0.2–1.4)	0.212	–	–
Central line associated infection	54 (12.7)	6 (9.5)	0.7 (0.3–1.8)	0.507	–	–
Urinary tract infection	7 (1.6)	1 (1.6)	1.0 (1.1–8.0)	1.000	–	–
Pneumonia	2 (0.5)	4 (6.3)	14.4 (2.6–80.2)	0.003	32.7 (5.1–208.5)	< 0.001
Others	9 (2.1)	0 (0.0)	0.9 (0.8–0.9)	0.374	–	–
Unknown	288 (67.6)	46 (73.0)	1.3 (0.7–2.3)	0.389	–	–
Pitt score, median (IQR)	1.3 (0.0–2.0)	4.1 (1.0–5.0)	1.5 (1.3–1.7)	< 0.001	1.5 (1.3–1.7)	< 0.001
Septic shock	73 (17.1)	29 (46.0)	4.1 (2.4–7.2)	< 0.001	–	–
Carba-NS	24 (5.6)	21 (33.3)	8.4 (4.3–16.3)	< 0.001	6.6 (3.0–14.8)	< 0.001
Inappropriate empiric antibiotics	27 (6.3)	13 (20.6)	3.8 (1.9–7.9)	< 0.001	–	–
Hospital stay, median						
Days from chemotherapy to GNB bacteremia, median (IQR)	14.2 (11.0–16.0)	18.0 (11.0–21.0)	1.1 (1.0–1.1)	< 0.001	–	–
Hospital days to GNB bacteremia, median (IQR)	21.2 (14.0–20.0)	40.3 (18.0–61.0)	1.0 (1.0–1.1)	< 0.001	–	–

*OR* odds ratio, *aOR* adjusted odds ratio, *CI* confidence interval, *SD* standard deviation, *VRE* vancomycin resistant enterococci, *ESBL* extended-spectrum β-lactamase-producing enterobacteriaceae, *CRPA* carbapenem resistant *Pseudomonas aeruginosa*, *CRAB* carbapenem resistant *Acinetobacter baumannii*, *GNB* gram negative bacilli, *IQR* interquartile range, *Carba-NS* carbapenem non-susceptible

Also, compared to the survival group, the attributed mortality group showed significantly higher percentages of carba-NS GNB bacteremia (17/43, 39.5% in attributed mortality vs. 24/426, 5.6% in survival; *P* < 0.001, see Additional file 1: Table S1). Carba-NS GNB bacteremia was independently associated with attributed mortality after adjusting with clinical characteristics (aOR: 9.9, 95%CI: 3.5–27.7, *P* < 0.001).

### Sensitivity analysis

To perform a subgroup analysis, we excluded all breakthrough GNB bacteremia cases that occurred during carbapenem use. Among these 459 cases, 20 (4.4%) were carba-NS and 439 (95.6%) were carba-S cases. Among the carba-NS cases, the most frequent carba-NS isolates were *Stenotrophomonas maltophilia* (8 cases, 40.0%), *Pseudomonas aeruginosa* (5 cases, 25.0%), and *Acinetobacter baumannii* (4 cases, 20.0%).

Univariate and multivariate analyses were performed to elucidate independent risk factors for carba-NS GNB bacteremia in this group of patients (see Additional file 1: Table S2). Independent risk factors for carba-NS GNB bacteremia without carbapenem exposure were the isolation of CRAB in the prior year and a time interval between chemotherapy and bacteremia. Carba-NS cases were also independently associated with in-hospital mortality in this subgroup (see Additional file 1: Table S3).

## Discussion

Poor clinical outcomes are known to be associated with delayed administration of the appropriate antibiotics for GNB bacteremia in patients with AML [7, 8]. Although carbapenems cover a broad spectrum of gram-negative bacteria, 9.2% of GNB bacteremia cases were due to carba-NS isolates in this study. Moreover, the clinical outcomes of these cases were independently worse than the outcomes in carba-S GNB cases. We found independent risk factors for carba-NS GNB bacteremia, including carbapenem use at bacteremia onset, the isolation of CRAB in the prior year, and a prolonged time interval between chemotherapy and the onset of bacteremia.

Although carbapenem use was the most important risk factor for carba-NS GNB bacteremia, 44.4% (20/45) of cases occurred without carbapenem administration. To reveal independent risk factors of carba-NS GNB bacteremia when carbapenems were not being administered, we performed an additional subgroup analysis. This analysis revealed that the recent isolation of a resistant organism and a prolonged interval between chemotherapy and bacteremia onset were independently associated with carba-NS GNB bacteremia. Therefore, when GNB bacteremia occurs in a patient with these risk factors, clinicians should consider the possibility of carba-NS pathogens, even when carbapenem was not being used.

The few previous studies that investigated risk factors for carba-NS GNB bacteremia included all hospitalized patients [9, 10]. The first retrospective study, which was conducted in Taiwan, revealed independent risk factors for carba-NS GNB bacteremia, including previous exposure to carbapenem, and a longer hospital stay before the development of bacteremia [9]. Another case-control study showed that breakthrough GNB bacteremia during carbapenem use was significantly associated with a longer hospital stay, the presence of hematologic malignancy, and a previous colonization of causative microorganisms [10]. Although these risk factors were similar to the results of the present study, our study specifically focused on patients with AML that underwent chemotherapy.

In this study, the major pathogens identified in carba-NS GNB bacteremia were *S. maltophilia*, *P. aeruginosa*, and *A. baumannii*. Similar to previous studies, *S. maltophilia* was the most frequently identified [9, 10, 17]. Since we did not find any clinical characteristics that were independently associated with those respective organisms (data not shown), it was difficult to suggest a specific antibiotic strategy. However, in patients with GNB bacteremia that have those risk factors, especially patients that are critically ill, it would be reasonable to add trimethoprim/sulfamethoxazole and/or colistin before knowing the results of antimicrobial susceptibility tests. Further studies are warranted to elucidate whether the empirical administration of antibiotics that target carba-NS organisms might have an impact on the clinical outcomes of GNB bacteremia.

Previous studies have shown that the clinical outcome of carba-NS GNB bacteremia was worse than that of carba-S GNB bacteremia [8, 9, 18, 19]. In this study, carba-NS GNB bacteremia was independently associated with in-hospital mortality after adjusting with clinical characteristics [18, 20]. In fact, inappropriate empirical antibiotic choices were observed significantly more often in the carba-NS group than in the carba-S group. It was previously shown that inappropriate, empirically selected antibiotic treatment was associated with in-hospital mortality [8]. Therefore, rapid antibiotic susceptibility tests that facilitate the selection of optimal agents within a short time might be an option for improving clinical outcomes of this critical condition [21, 22].

This study had some limitations. First, it was a retrospective study with a limited number of carba-NS GNB bacteremia cases. Therefore, the results should be validated in a larger prospective cohort. Second, this study was conducted in a single tertiary-care hospital. Since circulating organisms and their antimicrobial susceptibility might vary from hospital to hospital, the results of the present study should be interpreted prudently in other institutions.

## Conclusion

We showed that, among patients with AML, the development of carba-NS GNB bacteremia during induction or consolidation chemotherapy was independently associated with a previous isolation of resistant organisms, a late onset of GNB bacteremia, and the use of carbapenem at onset. The clinical outcomes of carba-NS GNB bacteremia were independently worse than those of carba-S GNB bacteremia. Carba-NS GNB organisms should be considered when selecting empirical antibiotics in patients with AML that have those risk factors.

## Supplementary information

**Additional file 1: Table S1.** Risk factors for gram negative bacilli bacteremia-attributed mortality. **Table S2.** Risk factors for carba-NS GNB bacteremia while not using carbapenem. **Table S3.** Risk factors for in-hospital mortality while not using carbapenem.

## Data Availability

The datasets used and/or analysed during the current study are available from the corresponding author on reasonable request.

## Abbreviations

GNB: Gram negative bacilli
AML: Acute myelogenous leukemia
Carba-NS: Carbapenem not susceptible
Carba-S: Carbapenem susceptible
VRE: Vancomycin-resistant enterococci
ESBL: Extended-spectrum β-lactamase-producing enterobacteriaceae
CRAB: Carbapenem-resistant *Acinetobacter baumannii*
aOR: Adjusted odds ratio
CI: Confidence interval
