# Supplementary material for: Risk factors for and clinical outcomes of carbapenem non-susceptible gram negative bacilli bacteremia in patients with acute myelogenous leukemia
Source: BMC Infect Dis. 2020 Jun 9;20:404. doi: 10.1186/s12879-020-05131-2 (PMC7282079; doi:10.1186/s12879-020-05131-2)
Supplement: Supplementary file 1 — Additional file 1: Table S1. Risk factors for gram negative bacilli bacteremia-attributed mortality. Table S2. Risk factors for carba-NS GNB bacteremia while not using carbapenem. Table S3. Risk factors for in-hospital mortality while not using carbapenem. [file 12879_2020_5131_MOESM1_ESM.docx]

**Table S1** Risk factors for gram negative bacilli bacteremia-attributed mortality in this study

| Variables | Survival  (N=426) | Attributed mortality  (N=43) | Univariate | | Multivariate | |
| --- | --- | --- | --- | --- | --- | --- |
|  |  |  | **OR (95%CI)** | ***P*** | **aOR (95%CI)** | ***P*** |
| Age, mean (± SD) | 50.9 (± 14.8) | 59.0 (± 12.7) | 1.0 (1.0-1.1) | 0.001 | - | - |
| Male | 250 (58.7) | 21 (48.8) | 0.7 (0.4-1.3) | 0.213 | - | - |
| Chemotherapy |  |  |  |  |  |  |
| induction or re-induction | 158 (37.1) | 29 (67.4) | 3.5 (1.8-6.8) | <0.001 | 2.6 (1.0-6.7) | 0.045 |
| consolidation | 268 (62.9) | 14 (32.6) | - | - | - | - |
| Diabetes mellitus | 113 (26.5) | 18 (41.9) | 2.0 (1.0-3.8) | 0.033 | 2.6 (1.1-6.6) | 0.037 |
| Isolation of resistant organism in the prior 1 year |  |  |  |  |  |  |
| VRE | 27 (6.3) | 14 (32.6) | 7.1 (3.4-15.1) | <0.001 | 7.0 (2.4-20.7) | <0.001 |
| ESBL | 21 (4.9) | 7 (16.3) | 3.8 (1.5-9.4) | 0.009 | - | - |
| CRPA | 8 (1.9) | 1 (2.3) | 1.2 (0.2-10.2) | 0.582 | - | - |
| CRAB | 4 (0.9) | 5 (11.6) | 13.9 (3.6-53.9) | <0.001 | - | - |
| History of GNB bacteremia in the prior 1 year | 136 (31.9) | 22 (51.2) | 2.2 (1.2-4.2) | 0.011 | - | - |
| Presence of preceding bacteremia during the hospitalization | 42 (9.9) | 13 (30.2) | 4.0 (2.0-8.2) | <0.001 | - | - |
| Primary foci of infection |  |  |  |  |  |  |
| Intra-abdominal infection | 66 (15.5) | 3 (7.0) | 0.4 (0.1-1.4) | 0.133 | - | - |
| Central line associated infection | 54 (12.7) | 3 (7.0) | 0.5 (0.2-1.7) | 0.276 | - | - |
| Urinary tract infection | 7 (1.6) | 0 (0.0) | 1.0 (0.9-0.9) | 1.000 | - | - |
| Pneumonia | 2 (0.5) | 3 (7.0) | 15.9 (2.6-98.0) | 0.006 | 61.2 (8.1-462.0) | <0.001 |
| Others | 9 (2.1) | 0 (0.0) | 0.9 (0.9-0.9) | 0.374 | - | - |
| Unknown | 288 (67.6) | 34 (79.1) | 1.8 (0.8-3.9) | 0.122 | - | - |
| Pitt score, median (IQR) | 1.3 (0.0–2.0) | 5.3 (1.0–9.0) | 1.8 (1.5-2.1) | <0.001 | 1.7 (1.5-2.1) | <0.001 |
| Septic shock | 73 (17.1) | 27 (62.8) | 8.2 (4.2-15.9) | <0.001 | - | - |
| Carba-NS | 24 (5.6) | 17 (39.5) | 11.0 (5.2-22.9) | <0.001 | 9.9 (3.5-27.7) | <0.001 |
| Inappropriate empiric antibiotics | 27 (6.3) | 11 (25.6) | 5.1 (2.3-11.2) | <0.001 | - | - |
| Hospital stay, median |  |  |  |  |  |  |
| Days from chemotherapy to GNB bacteremia, median (IQR) | 14.2 (11.0-16.0) | 18.3 (12.0-21.0) | 1.1 (1.0-1.1) | <0.001 | - | - |
| Hospital days to GNB bacteremia, median (IQR) | 21.2 (14.0-20.0) | 35.5 (17.0-55.0) | 1.0 (1.0-1.0) | <0.001 | - | - |

*OR* odds ratio, *aOR* adjusted odds ratio, *CI* confidence interval, *SD* standard deviation, *VRE* vancomycin resistant enterococci, *ESBL* extended-spectrum β-lactamase-producing enterobacteriaceae, *CRPA* carbapenem resistant *Pseudomonas aeruginosa*, *CRAB* carbapenem resistant *Acinetobacter baumannii*, *GNB* gram negative bacilli, *IQR* interquartile range, *Carba-NS* carbapenem non-susceptible

**Table S2** Risk factors for carba-NS GNB bacteremia while not using carbapenem in this study

| Variables | Carba-S  (n=439) | Carba-NS  (n=20) | Univariate | | | Multivariate | | |
| --- | --- | --- | --- | --- | --- | --- | --- | --- |
|  |  |  | **OR (95%CI)** | ***P*** | | **aOR (95%CI)** | | ***P*** |
| Age, mean (± SD) | 51.0 (± 14.8) | 56.6 (± 16.2) | 1.0 (1.0-1.1) | | 0.099 | - | - | |
| Male | 259 (59.0) | 9 (45.0) | 0.6 (0.2-1.4) | | 0.214 | - | - | |
| Chemotherapy |  |  |  | |  |  |  | |
| induction or re-induction | 167 (38.0) | 13 (65.0) | 3.0 (1.2-7.7) | | 0.016 | - | - | |
| consolidation | 272 (62.0) | 7 (35.0) | - | | - | - | - | |
| Diabetes mellitus | 121 (27.6) | 7 (35.0) | 1.4 (0.6-3.6) | | 0.468 | - | - | |
| Isolation of resistant organism in the prior 1 year |  |  |  | |  |  |  | |
| VRE | 32 (7.3) | 2 (10.0) | 1.4 (0.3-6.4) | | 0.654 | - | - | |
| ESBL | 22 (5.0) | 3 (15.0) | 3.3 (0.9-12.3) | | 0.088 | - | - | |
| CRPA | 8 (1.8) | 1 (5.0) | 2.8 (0.3-23.8) | | 0.333 | - | - | |
| CRAB | 3 (0.7) | 3 (15.0) | 25.6 (4.8-136.5) | | 0.001 | 19.3 (3.4-110.4) | 0.001 | |
| History of GNB bacteremia in the prior 1 year | 143 (32.6) | 8 (40.0) | 1.4 (0.6-3.5) | | 0.489 | - | - | |
| Presence of preceding bacteremia during the hospitalization | 42 (9.6) | 6 (30.0) | 4.1 (1.5-11.1) | | 0.012 | - | - | |
| Primary foci of infection |  |  |  | |  |  |  | |
| Intra-abdominal infection | 66 (15.0) | 1 (5.0) | 0.3 (0.0-2.3) | | 0.334 | - | - | |
| Central line associated infection | 51 (11.6) | 4 (20.0) | 1.9 (0.6-6.0) | | 0.276 | - | - | |
| Urinary tract infection | 8 (1.8) | 0 (0.0) | 1.0 (0.9-1.0) | | 1.000 | - | - | |
| Pneumonia | 6 (1.4) | 0 (0.0) | 1.0 (0.9-1.0) | | 1.000 | - | - | |
| Others | 9 (2.1) | 0 (0.0) | 1.0 (0.9-1.0) | | 1.000 | - | - | |
| Unknown | 299 (68.1) | 15 (75.0) | 1.4 (0.5-3.9) | | 0.517 | - | - | |
| Pitt score, median (IQR) | 1.6 (0.0-2.0) | 1.3 (0.0-1.0) | 0.9 (0.7-1.2) | | 0.464 | - | - | |
| Septic shock | 90 (20.5) | 1 (5.0) | 0.2 (0.0-1.5) | | 0.146 | - | - | |
| Inappropriate empiric antibiotics | 12 (2.7) | 13 (65.0) | 66.1 (22.4-195.2) | | <0.001 | - | - | |
| Hospital stay, median |  |  |  | |  |  |  | |
| Days from chemotherapy to GNB bacteremia, median (IQR) | 14.0 (11.0-16.0) | 19.6 (12.5-24.3) | 1.1 (1.1-1.2) | | <0.001 | 1.1 (1.1-1.2) | <0.001 | |
| Hospital days to GNB bacteremia, median (IQR) | 22.1 (14.0-20.0) | 33.7 (22.0-42.0) | 1.0 (1.0-1.0) | | 0.007 | - | - | |

*Carba-S* carbapenem susceptible, *Carba-NS* carbapenem non-susceptible, *OR* odds ratio, *aOR* adjusted odds ratio, *CI* confidence interval, *SD* standard deviation, *VRE* vancomycin resistant enterococci, *ESBL* extended-spectrum β-lactamase-producing enterobacteriaceae, *CRPA* carbapenem resistant *Pseudomonas aeruginosa*, *CRAB* carbapenem resistant *Acinetobacter baumannii*, *GNB* gram negative bacilli, *IQR* interquartile range

**Table S3** Risk factors for in-hospital mortality while not using carbapenem in this study

| Variables | Survival  (N=414) | In-hospital mortality  (N=45) | Univariate | | | Multivariate | | |
| --- | --- | --- | --- | --- | --- | --- | --- | --- |
|  |  |  | **OR (95%CI)** | ***P*** | | **aOR (95%CI)** | | ***P*** |
| Age, mean (± SD) | 50.9 (± 14.9) | 53.9 (± 14.8) | 1.0 (1.0-1.0) | | 0.196 | - | - | |
| Male | 244 (58.9) | 24 (53.3) | 0.8 (0.4-1.5) | | 0.525 | - | - | |
| Chemotherapy |  |  |  | |  |  |  | |
| induction or re-induction | 148 (35.7) | 32 (71.1) | 4.4 (2.3-8.7) | | <0.001 | 4.6 (2.1-10.2) | <0.001 | |
| consolidation | 266 (64.3) | 13 (28.9) | - | | - | - | - | |
| Diabetes mellitus | 109 (26.3) | 19 (42.2) | 2.0 (1.1-3.8) | | 0.034 | - | - | |
| Isolation of resistant organism in the prior 1 year |  |  |  | |  |  |  | |
| VRE | 27 (6.5) | 7 (15.6) | 2.6 (1.1-6.5) | | 0.038 | - | - | |
| ESBL | 21 (5.1) | 4 (8.9) | 1.8 (0.6-5.6) | | 0.292 | - | - | |
| CRPA | 6 (1.4) | 3 (6.7) | 4.9 (1.2-20.1) | | 0.049 | 8.2 (1.7-40.9) | 0.010 | |
| CRAB | 4 (1.0) | 2 (4.4) | 4.8 (0.8-26.8) | | 0.109 | - | - | |
| History of GNB bacteremia in the prior 1 year | 130 (31.4) | 21 (46.7) | 1.9 (1.0-3.6) | | 0.038 | 2.3 (1.1-5.0) | 0.037 | |
| Presence of preceding bacteremia during the hospitalization | 37 (8.9) | 11 (24.4) | 3.3 (1.5-7.0) | | 0.003 | - | - | |
| Primary foci of infection |  |  |  | |  |  |  | |
| Intra-abdominal infection | 62 (15.0) | 5 (11.1) | 0.7 (0.3-1.9) | | 0.486 | - | - | |
| Central line associated infection | 50 (12.1) | 4 (8.9) | 0.7 (0.2-2.0) | | 0.528 | - | - | |
| Urinary tract infection | 7 (1.7) | 1 (2.2) | 1.3 (0.2-11.0) | | 0.565 | - | - | |
| Pneumonia | 2 (0.5) | 4 (8.9) | 20.1 (3.6-113.1) | | 0.001 | 36.5 (5.3-252.3) | <0.001 | |
| Others | 10 (2.4) | 0 (0.0) | 0.9 (0.9-0.9) | | 0.608 | - | - | |
| Unknown | 283 (68.4) | 31 (68.9) | 1.0 (0.5-2.0) | | 1.000 | - | - | |
| Pitt score, median (IQR) | 1.3 (0.0-2.0) | 4.0 (0.5-6.5) | 1.5 (1.3-1.7) | | <0.001 | 1.5 (1.3-1.7) | <0.001 | |
| Septic shock | 72 (17.4) | 19 (42.2) | 3.5 (1.8-6.6) | | <0.001 | - | - | |
| Carba-NS | 15 (3.6) | 5 (11.1) | 3.3 (1.1-9.6) | | 0.037 | 3.5 (1.0-12.1) | 0.046 | |
| Inappropriate empiric antibiotics | 20 (4.8) | 5 (11.1) | 2.5 (0.9-6.9) | | 0.086 | - | - | |
| Hospital stay, median |  |  |  | |  |  |  | |
| Days from chemotherapy to GNB bacteremia, median (IQR) | 14.1 (11.0-16.0) | 15.5 (11.0-19.0) | 1.0 (1.0-1.1) | | 0.090 | - | - | |
| Hospital days to GNB bacteremia, median (IQR) | 20.6 (14.0-20.0) | 41.0 (16.5-64.5) | 1.0 (1.0-1.1) | | <0.001 | - | - | |

*OR* odds ratio, *aOR* adjusted odds ratio, *CI* confidence interval, *SD* standard deviation, *VRE* vancomycin resistant enterococci, *ESBL* extended-spectrum β-lactamase-producing enterobacteriaceae, *CRPA* carbapenem resistant *Pseudomonas aeruginosa*, *CRAB* carbapenem resistant *Acinetobacter baumannii*, *GNB* gram negative bacilli, *IQR* interquartile range, *Carba-NS* carbapenem non-susceptible
